# Supplementary material for: Vitamin D Modified DSS-Induced Colitis in Mice via STING Signaling Pathway
Source: Biology (Basel). 2025 Jun 18;14(6):715. doi: 10.3390/biology14060715 (PMC12190092; doi:10.3390/biology14060715)
Supplement: Supplementary file 1 [file biology-14-00715-s001.zip › Supplementary Materials S1: 16s-report of gut microbiota/Groups/diff_analysis/UnivarTestClass/figure/boxplot/top10log.pdf]

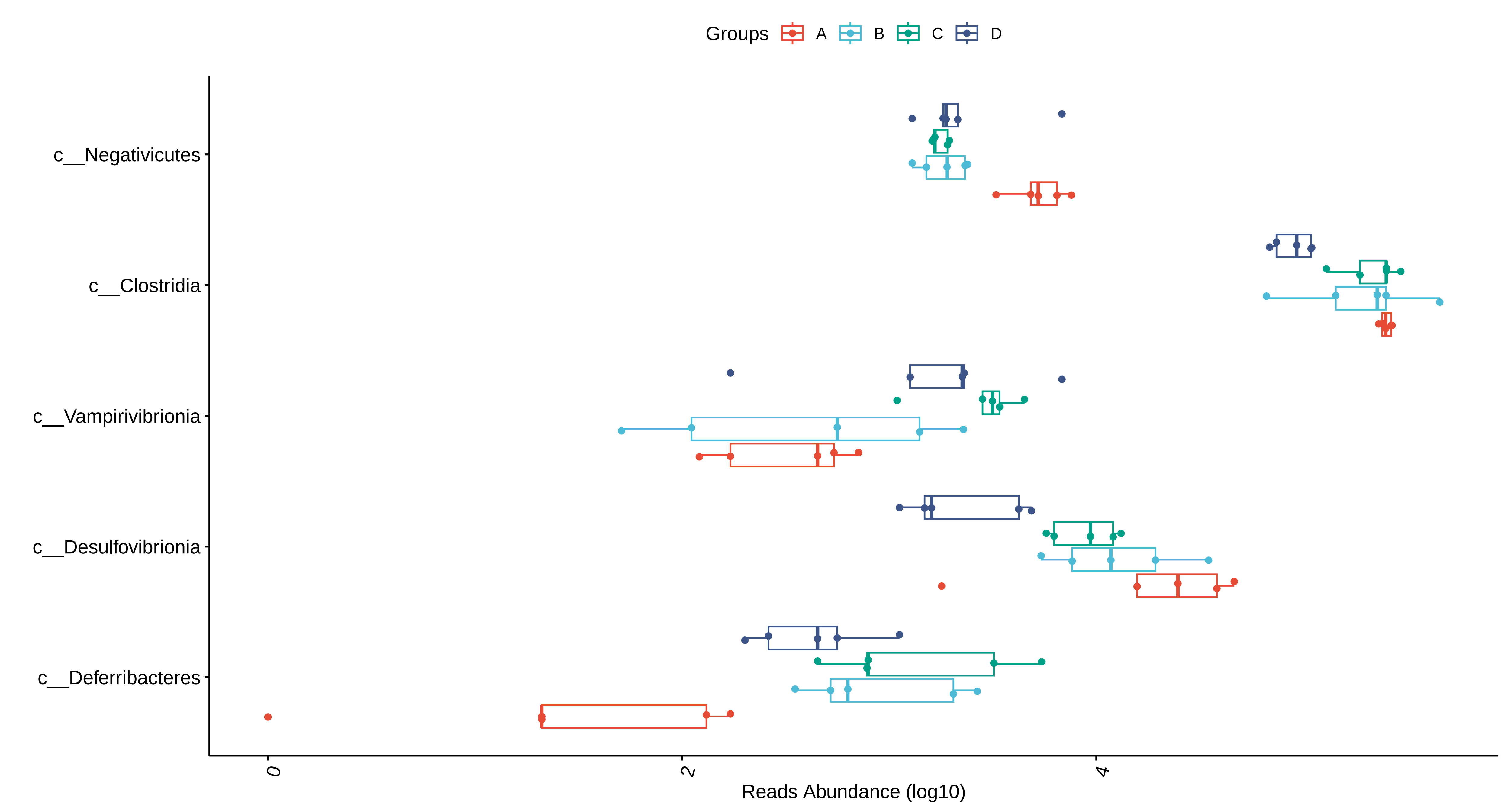

Groups
A
B
C
D
c__Negativicutes
c__Clostridia
c__Vampirivibrionia
c__Desulfovibrionia
c__Deferribacteres
0
2
4
Reads Abundance (log10)
